# Comparative Evaluation of Condylar Guidance Angles Measured Using Arcon and Non-Arcon Articulators and Panoramic Radiographs—A Systematic Review and Meta-Analysis

**DOI:** 10.3390/life13061352

**Published:** 2023-06-08

**Authors:** Amjad Obaid Aljohani, Mohammed Ghazi Sghaireen, Muhammad Abbas, Bader Kureyem Alzarea, Kumar Chandan Srivastava, Deepti Shrivastava, Rakhi Issrani, Merin Mathew, Ahmed Hamoud L Alsharari, Mohammed Ali D. Alsharari, Naif Abdulrahman Aljunaydi, Saif Alanazi, Mosheri Muslem S. Alsharari, Mohammad Khursheed Alam

**Affiliations:** 1 Department of Prosthodontics, College of Dentistry, Jouf University, Sakaka 72345, Saudi Arabia; dr.amjad.johani@jodent.org (A.O.A.); mayali@ju.edu.sa (M.A.); bkzarea@jodent.org (B.K.A.); dr.merin.mathew@jodent.org (M.M.); 2 Division of Oral Medicine & Maxillofacial Radiology, Department of Oral & Maxillofacial Surgery & Diagnostic Sciences, College of Dentistry, Jouf University, Sakaka 72345, Saudi Arabia; 3 Preventive Dentistry Department, Division of Periodontics, College of Dentistry, Jouf University, Sakaka 72345, Saudi Arabia; sdeepti20@gmail.com (D.S.); dr.rakhi.issrani@jodent.org (R.I.); 4 Department of Periodontics, Saveetha Dental College and Hospitals, Saveetha Institute of Medical and Technical Sciences, Saveetha University, Chennai 602105, India; 5 Dental Intern, College of Dentistry, Jouf University, Sakaka 72345, Saudi Arabia; ahsharari@gmail.com (A.H.L.A.); mohmmad.123678@gmail.com (M.A.D.A.); dr.naifaljunaydi@gmail.com (N.A.A.); saif.alanazi1122@gmail.com (S.A.); mosheri1417@gmail.com (M.M.S.A.); 6 Orthodontic Division, Preventive Dentistry Department, College of Dentistry, Jouf University, Sakaka 72345, Saudi Arabia; mkalam@ju.edu.sa; 7 Department of Dental Research Cell, Saveetha Institute of Medical and Technical Sciences, Saveetha Dental College and Hospitals, Chennai 602105, India; 8 Department of Public Health, Faculty of Allied Health Sciences, Daffodil international University, Dhaka 1216, Bangladesh

**Keywords:** temporomandibular joint, arcon articulator, condylar guidance angle, non-arcon articulator, panoramic radiographs, radiographic examination

## Abstract

The condylar guidance value (CGV) measurement constitutes an important part of a holistic prosthodontic treatment plan, with horizontal CGVs (HCGVs) and lateral CGVs (LCGVs) being two of the most prominently recognized. This systematic review aimed at evaluating the efficacy of two different types of CGV measurement protocols—articulators (both arcon and non-arcon) and panoramic radiographs. Additionally, it attempts to determine which of the mentioned methods performs better across several parameters. Several important web databases were searched using search terms derived from medical subject headings (MeSH), using keywords linked to “Arcon articulator”, “Condylar guidance angle”, “non-arcon articulator”, “Panoramic x-ray” and “Radiographic examination”, which constituted the first step in the study selection strategy. After completion, the search strategy which initially turned up to 831 papers, eventually ended up with 13 studies. The review and subsequent meta-analysis revealed that panoramic radiographs had noticeably greater efficacy in terms of the CGVs as compared to the articulators in the majority of the studies. Within the articulators, the arcon types recorded slightly higher CGVs than the non-arcon variety owing to the precision of jaw movement simulation in the former. However, further studies are required to validate these findings and establish more precise guidelines for the use of CGV measurement protocols in prosthodontic practice.

## 1. Introduction

One of the most common methods for measuring condylar guidance values (CGVs) is through panoramic radiographs [1,2,3]. Panoramic radiographs are two-dimensional images of the entire jaw and provide a panoramic view of the maxilla and mandible [4]. They allow for the measurement of the angle between the occlusal plane and the condylar path inclination [5]. The average CGVs range from 30 to 60 degrees, with higher values indicating a steeper condylar path. The CGVs are useful in determining the treatment plan for prosthodontic patients including the use of orthognathic surgery to correct severe jaw discrepancies. 

An articulator is a mechanical device that simulates the movement of the jaws and teeth, allowing the prosthodontist to accurately diagnose and plan prosthodontic treatment [6,7]. There are different types of articulators: simple-hinge or plane-line articulators, fixed condylar path (mean-value) articulators, and adjustable articulators. The adjustable articulators can be classified into semi-adjustable and fully adjustable groups. The fully adjustable articulators can be adjusted to simulate a wide range of movements of the jaw, including lateral and protrusive movements. The semi-adjustable articulators are designed to reproduce a fixed relationship between the maxilla and the mandible, and it can be classified into two types: arcon and non-arcon [8]. 

Arcon articulators have a condylar ball and socket mechanism that mimics the natural movement of the jaw. They are named after the German manufacturer who first developed this type of articulator. In an arcon articulator, the lower member, which represents the patient’s mandible, has two metal balls attached to it that fit into the corresponding sockets on the upper member, which represents the maxilla [8]. The arcon articulator allows easy adjustment of the occlusal plane, inclination of the occlusal plane, and lateral and protrusive movements of the mandible. Accuracy and reliability are the main advantages of arcon articulators. They closely mimic the natural movement of the jaw and provide a stable platform for prosthodontic diagnosis and treatment planning [9]. They are also highly durable and long-lasting. 

Non-arcon articulators do have a condylar ball and socket mechanism but the condyle is located in the upper member of the articulator. Instead, they use a hinge mechanism to simulate the movement of the jaw. They are less expensive than arcon articulators and are often used in dental schools and clinics that cannot afford expensive arcon articulators [7]. The main disadvantage of non-arcon articulators is their limited accuracy. They do not mimic the natural movement of the jaw as closely as arcon articulators and may not provide as stable a platform for prosthodontic diagnosis and treatment planning. However, they are still useful tools for many prosthodontic procedures, particularly in clinics and dental schools with limited resources [7].

Arcon and non-arcon articulators are important tools in prosthodontics that allow accurate diagnosis and treatment planning. The choice of articulator depends on the needs and resources of the individual prosthodontist or clinic [8].

Condylar guidance values (CGVs) are an essential parameter in the diagnosis and treatment planning of prosthodontic patients. They refer to the angle formed between the horizontal plane and the condylar path inclination during protrusive movement over the posterior slope of the articular eminence. This angle helps in determining the movement of the mandible during various functions such as chewing and speaking. These values can be measured using various methods, including clinical examination, radiographs, and digital imaging techniques.

Panoramic radiographs are commonly used to measure CGVs and help in determining the movement of the mandible during various functions such as chewing and speaking [10]. There are two different CGVs that are used in prosthodontics, including the horizontal condylar guidance value (HCGV), the lateral condylar guidance value (LCGV), protrusive guidance, and immediate lateral translation. The Bennett angle is the angle formed between the sagittal plane and condylar path inclination on the non-working side during lateral movement. It represents the lateral movement of the mandible during chewing and speaking. The average Bennett angle is around 15 degrees, with higher values indicating a more lateral mandibular movement [11]. The Immediate Lateral Translation (ILT) is the amount of lateral movement of the mandible during the initial opening of the mouth. This movement is important in the diagnosis and treatment planning of patients with temporomandibular joint (TMJ) disorders and during the fabrication of prosthesis. The average ILT is around 1.5 to 2.5 mm. The average protrusive guidance angle is around 30 degrees. 

Measuring these angles can be conducted through various methods, including clinical and laboratory examination, radiographs, and digital imaging techniques [11]. However, the cone beam-computed tomography is becoming popular, with wider applications such as the identification of osteoporosis [12] and the tracing of inferior alveolar nerve canal [13]; nonetheless, panoramic radiographs still remain the most common method for measuring CGVs [14,15]. The CGVs also play a significant role in the design and construction of occlusal splints, which are used to treat patients with TMJ disorders [16,17]. So, this systematic review and meta-analysis aimed to determine the accuracy of arcon and non-arcon articulators in measuring condylar guidance angles compared to panoramic radiographs. It also attempts to compare the differences in condylar guidance angle measurements between arcon and non-arcon articulators and panoramic radiographs. The secondary objectives include the identification of the factors affecting the accuracy of condylar guidance angle measurements using arcon and non-arcon articulators and panoramic radiographs. Additionally, this review attempts to assess the reliability and reproducibility of condylar guidance angle measurements using arcon and non-arcon articulators and panoramic radiographs.

## 2. Materials and Methods

### 2.1. Protocol and Research Framework

The current systematic review was registered on the International Prospective Register of Systematic Reviews (PROSPERO; registration number: CRD42023404427. The PICO (Population, Intervention, Comparison, Outcome) strategy for this study can be summarized as follows: The population of interest included patients or individuals who required evaluation of condylar guidance angles. The intervention under investigation was the measurement of condylar guidance angles using arcon articulators. The comparison involved the measurement of condylar guidance angles using non-arcon articulators. Additionally, the outcome of interest was the comparison of condylar guidance angles obtained from panoramic radiographs in relation to arcon and non-arcon articulators. The research question addressed in this study was whether there is any difference in the measurement of condylar guidance angles when using arcon and non-arcon articulators, as well as panoramic radiographs. 

The study followed a well-defined framework to ensure a rigorous and comprehensive analysis. Initially, an extensive search was conducted across multiple electronic databases and relevant sources were manually searched to identify eligible studies. During the study selection phase, pre-defined inclusion and exclusion criteria were applied to select studies that directly compared the measurement of condylar guidance angles using both arcon and non-arcon articulators, incorporating panoramic radiographs. Data extraction involved collecting relevant information such as study characteristics, participant demographics, and condylar guidance angle values obtained through different techniques. The quality assessment phase involved evaluating the methodological rigor and risk of bias in the included studies. A meta-analysis was then performed to synthesize the condylar guidance angle measurements from the selected studies, utilizing appropriate statistical methods to calculate pooled effect estimates and associated confidence intervals. Heterogeneity among the studies was assessed, and subgroup and sensitivity analyses were conducted to explore potential sources of heterogeneity.

### 2.2. Database Search Protocol

Following databases were searched using MeSH keywords for the extraction of relevant papers for this review:

• PubMed: ((systematic review[Title/Abstract] OR meta-analysis[Title/Abstract]) AND (“condylar guidance”[MeSH Terms] OR “condylar guidance”[Title/Abstract]) AND (“arcon articulator”[MeSH Terms] OR “arcon articulator”[Title/Abstract]) AND (“non-arcon articulator”[MeSH Terms] OR “non-arcon articulator”[Title/Abstract]) AND (“panoramic radiography”[MeSH Terms] OR “panoramic radiography”[Title/Abstract]));
• Google Scholar: “systematic review” OR “meta-analysis” AND “condylar guidance” AND (“arcon articulator” OR “non-arcon articulator”) AND “panoramic radiography”;
• Web of Science: TS = (“systematic review” OR “meta-analysis”) AND TS = (“condylar guidance” AND (“arcon articulator” OR “non-arcon articulator”)) AND TS = (“panoramic radiography”);
• Scopus: TITLE-ABS-KEY (“systematic review” OR “meta-analysis”) AND TITLE-ABS-KEY(“condylar guidance” AND (“arcon articulator” OR “non-arcon articulator”)) AND TITLE-ABS-KEY(“panoramic radiography”);
• EMBASE: (“condylar guidance angles” OR “condylar inclination” OR “condylar path” OR “mandibular movement”) AND (“arcon articulator” OR “arcon condylar guidance” OR “arcon condylar inclination” OR “arcon condylar path”) AND (“non-arcon articulator” OR “non-arcon condylar guidance” OR “non-arcon condylar inclination” OR “non-arcon condylar path”) AND (“panoramic radiograph” OR “orthopantomogram” OR “OPG”);
• LILACS: (“condylar guidance angles” OR “condylar inclination” OR “condylar path” OR “mandibular movement”) AND (“arcon articulator” OR “Arcon Condylar Guidance” OR “arcon condylar inclination” OR “arcon condylar path”) AND (“non-arcon articulator” OR “non-arcon condylar guidance” OR “non-arcon condylar inclination” OR “non-arcon condylar path”) AND (“panoramic radiograph” OR “Orthopantomogram” OR “OPG”);
• DOSS: (“condylar guidance angles” OR “condylar inclination” OR “condylar path” OR “mandibular movement”) AND (“arcon articulator” OR “arcon condylar guidance” OR “arcon condylar inclination” OR “arcon condylar path”) AND (“non-arcon articulator” OR “non-arcon condylar guidance” OR “non-arcon condylar inclination” OR “non-arcon condylar path”) AND (“panoramic radiograph” OR “orthopantomogram” OR “OPG”);
• Cochrane: (“condylar guidance angles” OR “condylar inclination” OR “condylar path” OR “mandibular movement”) AND (“arcon articulator” OR “Arcon Condylar Guidance” OR “Arcon condylar inclination” OR “arcon condylar path”) AND (“non-arcon articulator” OR “non-arcon condylar guidance” OR “non-arcon condylar inclination” OR “non-arcon condylar path”) AND (“panoramic radiograph” OR “Orthopantomogram” OR “OPG”).

### 2.3. Inclusion Criteria

• Studies that evaluated the efficacy of two different types of CGV measurement protocols—articulators (both arcon and non-arcon) and panoramic radiographs;
• Studies that compared the efficacy of the two CGV measurement protocols across several parameters;
• Studies that were in accordance with the search terms derived from MeSH-linked keywords such as “arcon articulator”, “condylar guidance angle”, “non-arcon articulator”, “panoramic radiographs “ and “radiographic examination”;
• Studies that provided information on the CGVs measured using both the articulators and panoramic radiographs;
• Studies published from 2011 to date.

### 2.4. Exclusion Criteria

• Studies that did not evaluate the efficacy of the two different types of CGV measurement protocols;
• Studies that did not compare the efficacy of the two CGV measurement protocols across several parameters;
• Studies that did not provide information on the CGVs measured using both the articulators and panoramic radiographs;
• Studies that did not reveal key information related to measurement of the type of CGV that was being assessed;
• Articles published before 2011;
• Studies that were case reports, seminar articles or thesis articles.

### 2.5. Reviewer Assessment and Bias Evaluation

**Figure 1:**
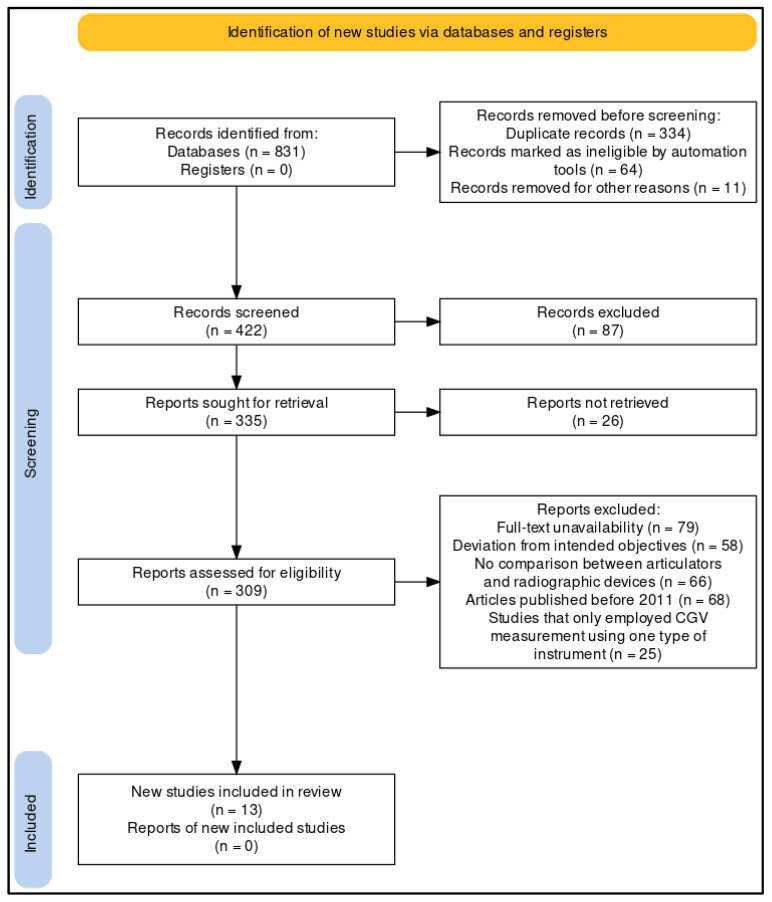
Study selection framework using the PRISMA protocol for this review.

**Figure 2:**
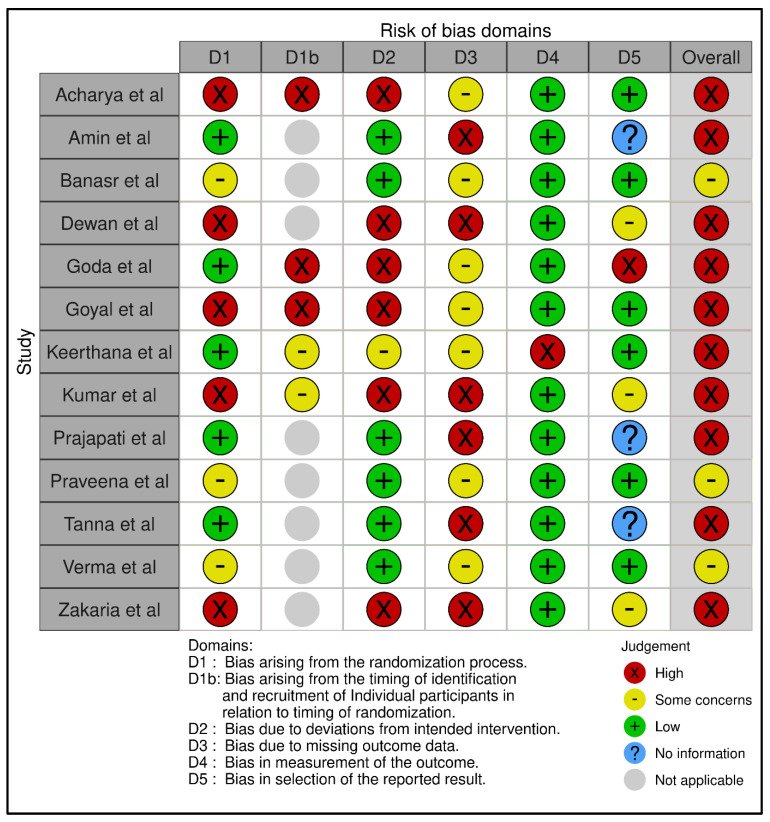
Bias assessment of individual studies using RoB-2 tool across five different domains [20,21,22,23,24,25,26,27,28,29,30,31,32].

Two independent reviewers who had expertise in prosthodontics and experience in conducting systematic reviews and meta-analyses were identified and recruited. Later, they independently evaluated the selected study to determine if it was relevant to the research question and meets the eligibility criteria. The PRISMA tool (Figure 1) was selected for the purpose of analyzing different types of studies and their relevance to this investigation, i.e., whether they were in accordance with the inclusion and exclusion criterion [18]. The reviewers evaluated the study’s objectives, methodology, and results. They also assessed its validity and reliability. They were also asked to document their evaluations independently and provide a summary of the reasons for their inclusion or exclusion of a certain study. Subsequently, they compared the evaluations and any discrepancies that arose were discussed and ironed out through discussion and consensus with another reviewer. They extracted the relevant data from the included studies, including the number of participants, type of measurement protocol, type of articulator used, type of panoramic radiograph used, CGV measurements, and any other relevant information. As for the bias assessment, the quality of studies being selected was evaluated using the RoB-2 tool (Figure 2). This tool assesses the risk of bias in studies by evaluating five different domains, the results of which were categorically separated into ‘low’, ‘moderate’, and ‘high’ risk of bias, respectively [19]. A meta-analysis of the assessed data was performed after the completion of the search strategy to evaluate the efficacy of the two different types of CGV measurement protocols, the results of which were interpreted, and conclusions were drawn.

### 2.6. Protocol for Meta-Analysis

Using the RevMan 5 software (Version 5.3, The Cochrane Collaboration), a meta-analysis was conducted. The objective was to determine the pooled effect size for the effectiveness of the two different types of articulators in comparison to the panoramic radiograph for measuring CGVs. Data on the type of measurement protocol and CGV type assessed were extracted from each included study, along with information on the mean age, parity, number of participants, type of measurement protocol, type of articulator used, and any other pertinent data (CIs). The risk ratio (RR) and risk differential (RD) were given with their own forest plot. In addition to the overall pooled estimate and its 95% CI, each forest plot also contained the summary estimate and its 95% CI for each research.

## 3. Results

In order to provide an updated review and assessment of CGVs, a keyword search was first conducted on various databases from the years 2011 to 2022. Following the implementation of the MeSH search strategy, initially a total of 831 papers were surfaced. Based on duplication and ineligibility by automation tools, the studies were filtered, resulting in a total of 422 papers. To make sure that only original papers were included, further screening of articles was conducted, which resulted in a total of 309 papers.

The titles and summaries of these 309 papers were scrutinized, and 296 additional papers that did not meet the inclusion/exclusion standards were ignored. Finally, we selected thirteen articles, which mainly contained in vivo and in vitro experiments, that met the required standards. These made up the final group of articles that were taken into account for the meta-analysis.

Five of the thirteen investigations [20,21,22,23,24] evaluated the measurement of sagittal CGV; the HCGV in was covered in seven [25,26,27,28,29,30,31]. (Table 1). The variety of studies that were reviewed leads one to believe that this significant issue may require a multi-disciplinary strategy to be addressed. OPG was used to produce panoramic radiographs in all of the studies that were considered for the evaluation. Individuals ranging in age from 18 to 75 were included in the research. Only three studies [22,29,31] considered CGV values acquired using a non-arcon articulator, while all studies [20,21,22,23,24,25,26,27,28,29,30,31,32] used an arcon articulator for CGV analysis. In the majority of studies [21,24,25,30,31,32], radiographs evaluated significantly higher values compared to the articulator, with panoramic X-rays evaluating higher CGVs compared to their mechanical measurement counterparts. Although in one study [31], males scored higher total mean values than females using both articulators for both HCGV and LCGV, with the arcon articulator obtaining higher overall mean values than the non-arcon articulator, gender correlation was not frequently sought.

**Figure 3:**
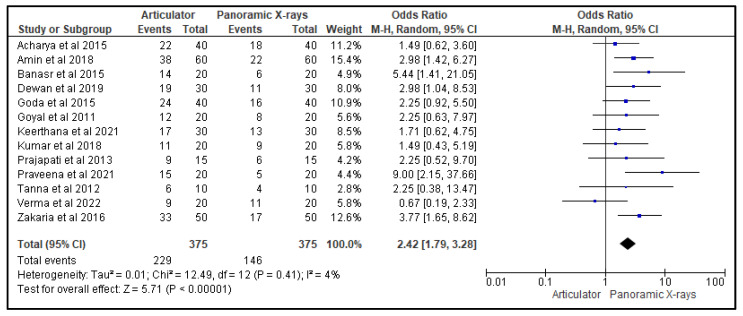
Forest plot representing the odds ratio of the efficacy of an articulator as compared to a panoramic X-ray for measuring CGVs in the 13 studies that were evaluated in this review [20,21,22,23,24,25,26,27,28,29,30,31,32].

**Figure 4:**
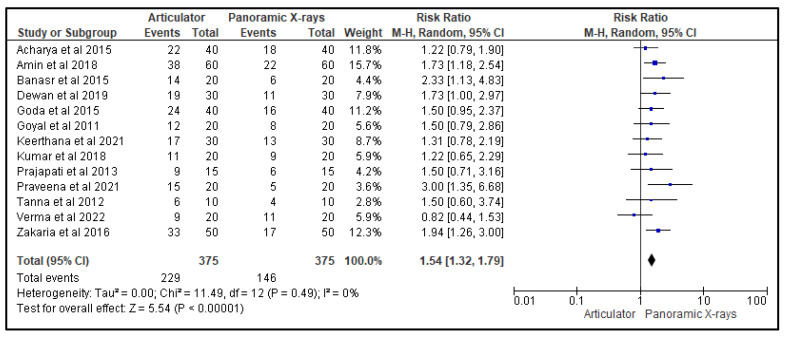
Forest plot representing the risk ratio of the efficacy of an articulator compared to a panoramic X-ray for measuring CGVs in the 13 studies that were evaluated in this review [20,21,22,23,24,25,26,27,28,29,30,31,32].

**Figure 5:**
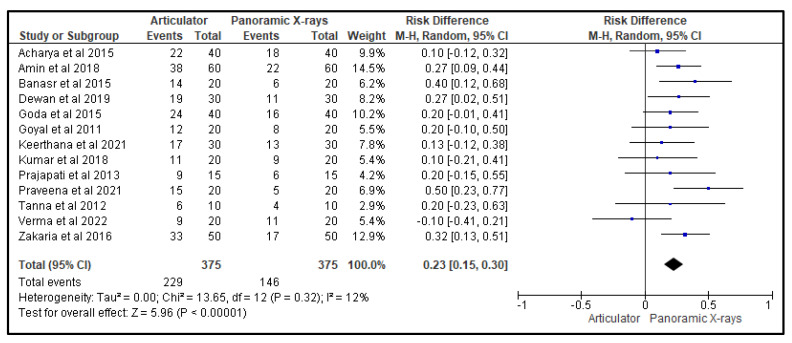
Forest plot representing the risk difference of the efficacy of an articulator compared to a panoramic X-ray for measuring CGVs in the 13 studies that were evaluated in this review [20,21,22,23,24,25,26,27,28,29,30,31,32].

Figure 3, Figure 4 and Figure 5 show, respectively, the forest plots from the 13 studies that were considered for the study. The data were entered into the RevMan 5 software after taking into account all relevant aspects of the papers, and three separate forest plots displaying the odds ratio, risk ratio, and risk difference related to the measurement of the CGV, using either the articulator or the panoramic X-ray that was noted in that study, were generated and evaluated. A random effects model with a 95% confidence interval was used in the meta-analysis. The overall number of events was the sample size for each paper.

Figure 3 is a forest plot that presents the odds ratio of the effectiveness of using an articulator versus a panoramic X-ray for measuring condylar guidance values (CGVs) in 13 studies. The random-effects model was used, and the confidence interval was set at 95%. The individual study results are represented as squares, with their size corresponding to the weight of the study, and the horizontal line across each square indicates the 95% confidence interval. The diamond at the bottom represents the summary effect estimate, with its width showing the confidence interval. The overall odds ratio was in favor of the panoramic X-ray, indicating that it was more effective in measuring CGVs compared to the articulators that were utilized in the studies.

Figure 4 is a forest plot that represents the risk ratio of the effectiveness of using an articulator versus a panoramic X-ray for measuring CGVs in the 13 studies evaluated in this review. The random-effects model was used, and the confidence interval was set at 95%. The individual study results are represented as squares, with their size corresponding to the weight of the study, and the horizontal line across each square indicates the 95% confidence interval. The diamond at the bottom represents the summary effect estimate, with its width showing the confidence interval. The overall odds ratio was also in favor of the panoramic X-ray, indicating that it was more effective in measuring CGVs compared to the articulators that were utilized in the studies.

Figure 5 is a forest plot that represents the risk difference of the effectiveness of using an articulator versus a panoramic X-ray for measuring CGVs in the 13 studies evaluated in this review. The random-effects model was used, and the confidence interval was set at 95%. The individual study results are represented as squares, with their size corresponding to the weight of the study, and the horizontal line across each square indicates the 95% confidence interval. The diamond at the bottom represents the summary effect estimate, with its width showing the confidence interval. The overall odds ratio was, again, in favor of the panoramic X-ray, indicating that it was more effective in measuring CGVs compared to the articulators that were utilized in the studies.

## 4. Discussion

Prosthodontics is a branch of dentistry which is concerned with the detection, prevention, and treatment of dental and facial abnormalities. Prosthodontic treatment involves fixed prosthesis, removable prosthesis, and maxillofacial prosthesis to improve the esthetics and rehabilitation of the oral cavity [33]. A holistic approach of prosthodontic treatment takes into account various factors including the CGVs. This measurement is an essential part of the prosthodontic treatment plan as it helps determine the ideal position of the mandible in relation to the maxilla during jaw movements. Cases with improper assessment of CGVs eventually lead to prothesis, which brings discomfort to oral functions, including the TMJ. Improper measurement of angle can also worsen the existing TMJ disorders affecting children, adults, and special patients [34,35]. It will eventually affect their quality of life [36]. In the current systematic review, the majority of the studies found that the panoramic radiographs had a noticeably greater efficacy in terms of the CGVs in contrast to the articulators. Within the articulators, the arcon recorded slightly higher CGVs than the non-arcon owing to the precision of jaw movement simulation. These findings provide important guidance for the prosthodontist to select the appropriate CGV measurement protocol. Therefore, the primary significance of the present systematic review lies in its contribution to the field of prosthodontics by evaluating the efficacy of two different CGV measurement protocols and providing important guidance for clinical practice. The findings have important implications in terms of improving the quality of care for patients; at the same time, they highlight the need for further research in this area.

The research methodologies that were observed in this systematic review were unique in a variety of ways. The condylar elements of the articulator are set in the clinical method so that they will replicate the inclinations close to the patient’s temporomandibular articulation using protrusive jaw relation. In the included experiments, the condylar guidance in semi-adjustable articulators was set using interocclusal protrusive wax records, Lucia jig, and gothic arch tracers. In studies using protrusive wax records, the amount of protrusion was maintained as constant for all patients at 6 mm, and the same protrusive records were used for programming the articulator. Hence, it is important to maintain protrusion distance as a constant as the HCGV changes with amount of protrusion [26,37]. The majority of studies used a Hanua Wide Vue semi-adjustable articulator after the protrusive jaw relation was determined, while a few studies used a whip mix semi-adjustable articulator to measure horizontal condylar inclination. To determine the horizontal condylar inclination, a reference line is used. The whip mix uses the nasion–porion as a reference plane; whereas Hanua articulators mount the cast in relation to the Frankfort horizontal plane, producing more precise angles [38,39]. Due to the substantial differences in the instruments, a slightly modified method for detecting SCGVs is inconsistent, lacks precision, and has lower levels of reproducibility [40,41]. The reasons for the compressed or deformed records include tipped casts due to incorrect cast adaptation, force exerted by the operator on the record, and records whose values have changed depending on the amount of protrusion, overjet, and overbite [1,41,42]. Additionally, because set inter-condylar distance and straight condylar pathway, the semi-adjustable articulators are unable to properly reconstruct the condylar movements [41].

One of the studies showed that intraoral recording methods produced lower values for condylar angles compared with the radiographic measurements. The man’s dynamic mandibular locomotor system appears to be incompatible with the fixed mechanical principles governing the movements of an adjustable articulator [25,43]. Hence, the diagnosis is now frequently made using CBCT, lateral cephalograms and panoramic radiographs. In the current review, significantly higher CGVs were found in panoramic radiographs than in protrusive interocclusal records. The findings of the review can be justified by the following rationales. In research by Gilboa et al. [44], it was shown that the panoramic radiographic method frequently produces a value that is higher than the actual value. In dry skulls, they found that the average sagittal CGV was seven degrees higher than its true anatomic contour. The resilient oral mucosa is depressed, and the inter-ridge distance is shortened when the occlusal rims are kept in a protruded mandibular position. It results in a narrower triangular wedge-shaped space between the posterior part of the occlusal rims. This space is similar to the Christensen’s space found in natural dentition and documented by protrusive interocclusal records [26,45,46].

Despite the fact that all of the included studies used the same reference line, there were differences in the results of the included patients. The observed variations can be due to patients’ head positioning, which causes parallax errors, the models of the panoramic machine, magnification variations, image distortions, overlapping of the anatomic structures such as mandibular notch, the coronoid process, and the zygomatic arch around TMJ [7,24,27,47,48,49].

Due to the fact that CBCT provides three-dimensional information for both sides without superimpositions, the glenoid fossa and other landmarks can be readily identified. The mean sagittal CGVs obtained from CBCT are marginally higher than those acquired from other techniques on both sides for dentate and edentulous individuals. Similar outcomes were found in the individual studies that were excluded from the review. For instance, in the study by Kumar et al. [50], condylar guidance values obtained from CBCT measurements were 5°–6° higher than those from protrusive occlusal records, while values obtained from clinical methods were attested in three studies mentioned in the literature [40,41,51]. The major benefit of CBCT over intraoral, panoramic, and cephalometric is the production of distinctive images that show 3D features. Cursor-driven measurement techniques give clinicians the ability to evaluate dimensions interactively and instantly. Additionally, the on-screen measurements are free from amplification and distortion.

Other benefits of CBCT include better image quality, smaller fields, faster scans, compatibility with different radiographic setups for image output, and simplicity of setup for minimal units in a general clinical setting. In edentulous and dentulous patients, these CBCT preferences may be used to identify the condylar position during dynamic registration and accurately locate the condyle [10,51]. The primary reason we did not include studies that used this measurement procedure was the high cost of the equipment, which is the main disadvantage of using CBCT.

Carossa et al. [52] provide another method; they use a jaw movement analyzer in conjunction with a robotic device to correctly record and duplicate mandibular movements. The system employs optoelectronic motion technology with markers attached to the jaw, allowing for quick and exact movement recording. Unlike earlier robotic systems, this one featured fewer mechanical components, which reduced tolerances and production costs. The results showed that the system accurately records and reproduces maxillomandibular relations in both static and dynamic settings. The authors stated that this robotic system represents a major advancement over existing analog and digital alternatives, providing cost savings, precision, and time-saving opportunities, and making it attractive for both clinical and research applications.

The study has a few limitations that need to be addressed. Firstly, the number of studies included in the review was relatively small, which may limit the generalizability of the findings. Secondly, the quality of the studies varied, and some of them had a high risk of bias, which may affect the validity of the results. Thirdly, the search strategy used in the study may have missed some relevant studies that could have contributed to the analysis. Finally, the study only evaluated the efficacy of two CGV measurement protocols and did not consider other potential measurement methods that may be available. Moreover, all the selected studies were from the South Asian region. Therefore, the results should be interpreted with caution, and further research is needed to confirm the findings and explore additional measurement protocols.

## 5. Conclusions

To conclude, this systematic review and meta-analysis evaluated the efficacy of two different types of CGV measurement protocols—articulators (both arcon and non-arcon) and panoramic radiographs. Moreover, it attempted to determine which performed better across several parameters. Based on the data from majority of the includes studies, the review found that panoramic radiographs had noticeably greater efficacy in terms of the CGVs. Within the articulators themselves, the arcon types recorded slightly higher CGVs than the non-arcon variety owing to the precision of jaw movement simulation in the former. This information can be useful for prosthodontic practitioners to make an informed decision about the type of CGV measurement protocol they choose to use for their patients. However, further studies are required to validate these findings and establish more precise guidelines for the use of CGV measurement protocols in prosthodontic practice.

## Figures and Tables

**Table 1:** Description of the variables evaluated in the 13 studies selected for review and subsequent meta-analysis.

Paper ID	Year	Region of Investigation	Study Design	Age Range (in Years)	Number of Participants	Type of Articulator Used	Type of CGV Assessed	Clinical Inference
Banasr et al. [20]	2015	Saudi Arabia	In-vitro	21–35	20	Arcon	Sagittal CGV	Similar values obtained from the articulator and radiographic images (little to no difference)
Dewan et al. [21]	2019	Saudi Arabia	In-vivo	20–40	30	Arcon	Sagittal CGV	Radiographs measured noticeably higher values as compared to the articulator
Goyal et al. [22]	2011	India	In-vivo	19–35	20	Arcon and Non-arcon	Sagittal CGV	Similar values obtained from the two types of articulators (little to no difference)
Kumar et al. [23]	2018	India	In-vitro	20–35	20	Arcon	Sagittal CGV	Similar values obtained from the articulator and radiographic images (little to no difference)
Tanna et al. [24]	2012	India	Prospective	-	10	Arcon	Sagittal CGV	Radiographs measured noticeably higher values as compared to the articulator
Acharya et al. [25]	2015	India	In-vivo	18–30, 40–75	40 (20 edentulous, 20 dentulous)	Arcon	HCGV	Radiographs measured noticeably higher values as compared to the articulator
Amin et al. [26]	2018	India	In-vivo	40–60	60	Arcon	HCGV	In dentulous subjects, statistically significant values were found using the articulator and radiographic method.
Goda et al. [27]	2015	India	In-vitro	20–30, 40–65	40 (20 edentulous, 20 dentulous)	Arcon	HCGV	Radiographs measured similar values as the articulator but only in edentulous individuals
Keerthana et al. [28]	2021	India	In-vivo	20–40	30	Arcon	HCGV	Similar values obtained from the articulator and radiographic images (little to no difference)
Prajapati et al. [29]	2013	India	In-vitro	20–30	15	Arcon and Non-arcon	zHCGV	Statistically insignificant values between the arcon-type, Non-arcon type and radiographic images were obtained across all parameters
Verma et al. [30]	2022	India	In-vivo	40–75	20	Arcon	HCGV	Radiographs measured higher values as compared to the articulator
Zakaria et al. [31]	2016	Iraq	In-vivo	30–65	50	Arcon and Non-arcon	HCGV and LCGV	Males scored higher total mean values than females using both articulators for both HCGV and LCGV, with the arcon articulator scoring higher overall mean values than the non-arcon articulator.
Praveena et al. [32]	2021	India	In-vitro	40–60	20	Arcon	LCGV	Radiographs measured noticeably higher values as compared to the articulator

## Data Availability

All data are available within the manuscript.
